# Gleanings

**Published:** 1896-05

**Authors:** 


### GLEANINGS.

Dr. Joseph M. Good, of Chattanooga, Tenn., reports the use of tetanus-antitoxin in two cases, one of which made a good recovery.

A Washington, D. C., canine died in the chair of a dentist who, at the solicitation of the owner of the dog, attempted to drill out a tooth, with a view of filling it.

Creolin finds one of its most useful fields in obstetrical practice among veterinarians. Its antiseptic properties, its emollient action on the parts, and where the latter have become dry and inflamed it facilitates manipulations, and serves thus a double purpose.

Warm creolin injections in constipation and impaction of the lower bowel of dogs aids wonderfully in remedying these conditions, and in very small dogs saves them from those painful manifestations that so often leave the animal in sore distress for days after the removal of the offending mass.

Good horses wanted by buyers, either for the track or road, are said to be worth 20 per cent. more than they were at this time last year.

